# Supplementary material for: Causal interplay between lactose intolerance and gut microbiota: a combined bidirectional Mendelian randomization and in vivo validation study
Source: Front Nutr. 2026 Jun 1;13:1803337. doi: 10.3389/fnut.2026.1803337 (PMC13265576; doi:10.3389/fnut.2026.1803337)
Supplement: Supplementary file 1 [file Data_Sheet_1.zip › supplementary materials/Forward/leave one out cross validation/ebi-a-GCST90027487.finngen_R12_E4_LACTONAS.pdf]

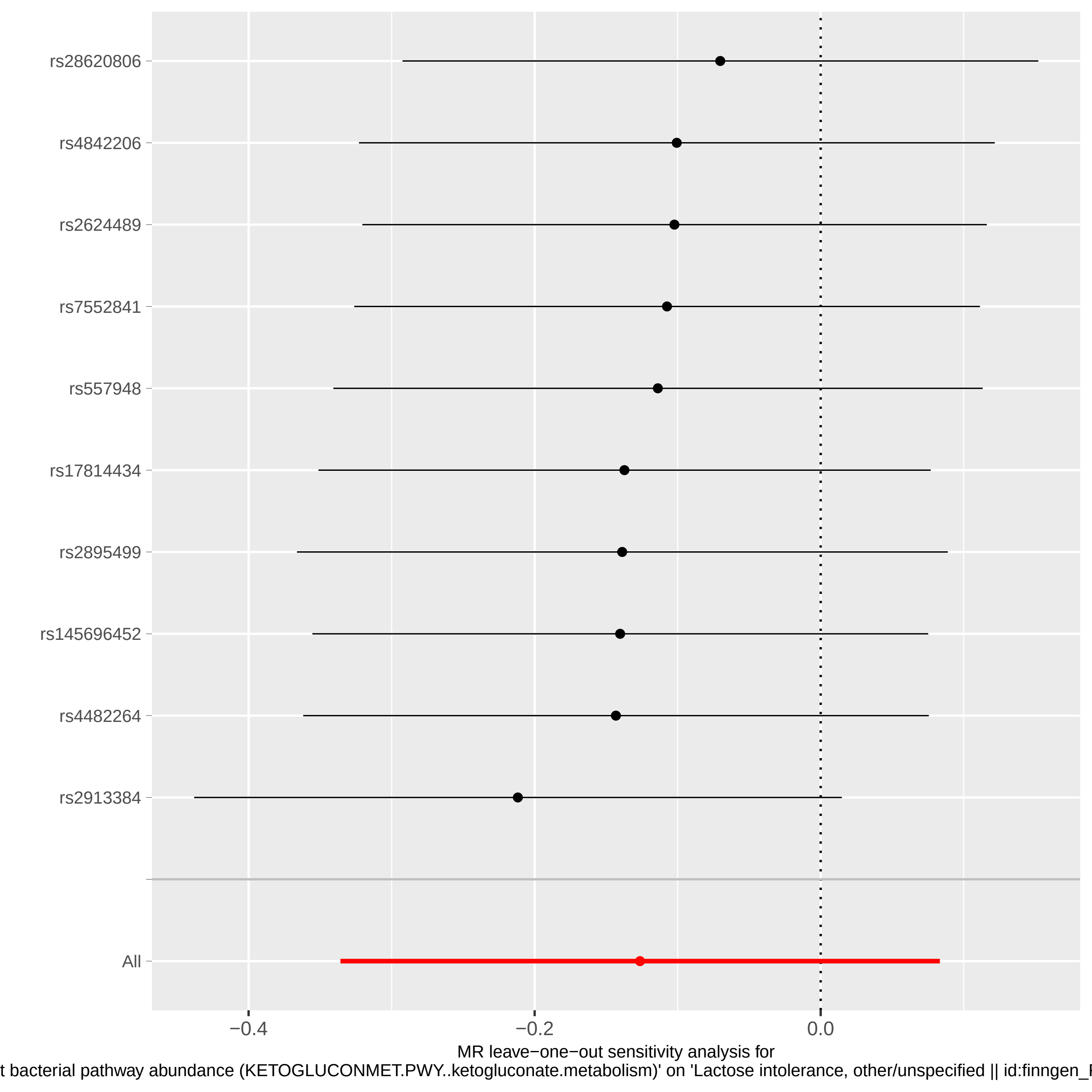
rs28620806
rs4842206
rs2624489
rs7552841
rs557948
rs17814434
rs2895499
rs145696452
rs4482264
rs2913384
All
−0.4
−0.2
0.0
MR leave−one−out sensitivity analysis for
t bacterial pathway abundance (KETOGLUCONMET.PWY..ketogluconate.metabolism)' on 'Lactose intolerance, other/unspecified || id:finngen_
